# Highly conductive and transparent copper nanowire electrodes on surface coated flexible and heat-sensitive substrates†

**DOI:** 10.1039/c7ra12738c

**Published:** 2018-01-09

**Authors:** Su Ding, Yanhong Tian, Jinting Jiu, Katsuaki Suganuma

**Affiliations:** State Key Laboratory of Advanced Welding and Joining, Harbin Institute of Technology Harbin 150001 China tianyh@hit.edu.cn; College of Materials and Environmental Engineering, Hangzhou Dianzi University Hangzhou 310036 China; The Institute of Scientific and Industrial Research (ISIR), Osaka University Osaka 5650871 Japan jiu@eco.sanken.osaka-u.ac.jp

## Abstract

Copper nanowire (CuNW) based flexible transparent electrodes have been extensively investigated due to their outstanding performances and low price. However, commonly used methods for processing CuNW transparent electrodes such as thermal annealing and photonic sintering inevitably damage the flexible substrates leading to low transmittance. Herein, a surface coating layer was demonstrated to protect the heat-sensitive polyethylene terephthalate (PET) polymer from being destroyed by the instantaneous high temperature during the photonic sintering process. The stable ceramic surface coating layer avoided the direct exposure of PET to intense light, further reduced the heat releasing to the bottom part of the PET, protecting the flexible PET base from destruction and ensuring high transparency for the CuNW transparent electrodes. A CuNW transparent electrode on surface coated PET (C-PET) substrates with a sheet resistance of 33 Ohm sq^−1^ and high transmittance of 82% has been successfully fabricated by the photonic sintering method using light intensity of 557 mJ cm^−2^ within several seconds in ambient conditions. The surface coating layers open a novel method to optimize the rapid photonic sintering technique for processing metal nanomaterials on heat-sensitive substrates.

## Abstract 2

The optoelectrical property of CuNW transparent electrodes on C-PET substrates was superior to that on N-PET because the surface coatings protected the destruction of PET polymer by the high-energy light during the photonic sintering process.

## Graphical abstract

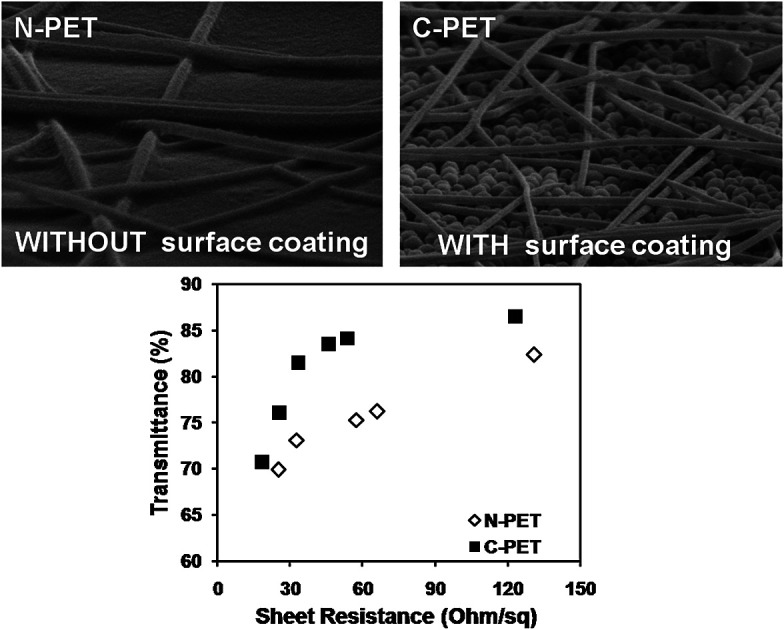

## 1 Introduction

Flexible electronics, as some of the most exciting and promising information technologies, have opened up new opportunities for prospective applications, such as foldable tablets and phones, bendable photovoltaic cells, bendable light emitting diodes (LEDs), and wearable sensors.^1–8^ Numerous efforts have been made to convert electronic components from traditional rigid silicon or printed circuit boards (PCB) onto flexible polymers, including polyethylene terephthalate (PET), or polyimide (PI) or polydimethylsiloxane (PDMS).^7,9,10^ To be compatible with the flexible substrates, the electronic components and packaged circuits have been developed with advanced and functional nanomaterials, which are easily assembled on bendable or irregular shaped substrates. Moreover, conventional photolithography and transfer printing processes are also replaced by new ink printing^11–13^ and sintering techniques,^1,14^ which are considered to cut the cost with high efficiency.

Transparent electrodes are essential component for most electronics, which are typically used as electrodes when a situation calls for low resistance electrical contacts without blocking light. Some applications, such as touch screens, often require a high transparency to view the underlying display in a wide range of visible light. Several nanomaterials have been demonstrated to be promising candidate for flexible transparent electrodes, including carbon nanotubes (CNTs),^15–17^ graphene,^11,18–20^ silver nanowires (AgNWs) and copper nanowires (CuNWs).^2,12,21–24^ CuNWs have attracted more and more attentions due to its high electrical conductivity, low price and abundance reserves. Wiley's group pioneered the fabrication of CuNW transparent conductors by similar annealing method in reducing atmosphere at high temperatures from 175–225 °C.^25,26^ The sheet resistance of the CuNW transparent electrodes was 100 Ohm sq^−1^ at 92% transparency after annealing.^26^ The contact resistance between nanowires was greatly reduced to achieve highly conductive electrodes by increasing the contact area and removing of surface residuals during the thermal annealing process. However, the high temperature (>200 °C) sintering step would destroy the heat-sensitive substrates, leading to an extreme low transparency for conductive films, which limits the applications in touch panel or display screen.

Recently, photonic sintering technique has turned up as promising method to treat metallic nanomaterials due to its extreme high speed, which was supposed to sinter metallic nanomaterials without destruction of polymer substrates.^10,27,28^ In fact, due to the extremely high energy of the incident light, the flexible polymer substrates were always heated or even burned.^29^ One solution is to improve the high temperature resistance of flexible substrates. For example, some Cu patterns also have been achieved with the light sintering on PI substrates because they can endure high temperature, which is also necessary for Cu sintering.^30,31^ However, PI substrates are yellowish and opaque below 500 nm wavelength, which limits their widespread applications as transparent devices. Another one is to adopt a multi-step process. Li *et al.*^32^ used a two-step sintering process to make Cu conductive pattern involving low temperature heat-welding under N_2_ atmosphere and subsequent flash light sinter reinforcement to refrain from destruction of heat-sensitive substrates. However, the two-step process was complicated and time-consuming, especially, a protective atmosphere was inevitable for Cu sintering in the heat-welding step. Although the photonic sintering technique is a fast, scalable and promising method to sinter metal nanomaterials, the destruction of flexible substrates should be avoided by simple procedure to enable the production of forthcoming flexible electronics on low-cost, transparent but heat-sensitive substrates, such as PET substrates.

Surface coating is an overlay protective covering on a substrate, which finds applications as thermal barrier, wear-resistant and corrosion resistance layers.^33,34^ For example, yttria stabilized zirconia (YSZ) coatings were used as the top layer of thermal barrier to lend thermal protection from hot gases in turbines and engines and lower the surface temperature of the base metal.^35,36^ The YSZ coatings provided a temperature drop of 100–200 °C due to their low thermal conductivity.^37^ Alumina layers were deposited on stainless steel substrate as wear and corrosion resistant coatings due to the high hardness, excellent wear resistance and high temperature stability.^38^ Magnesium oxide (MgO)/zirconium oxide (ZrO) duplex-layer has been prepared on AZ91D magnesium alloy as a protective coating against corrosion in sodium chloride (NaCl).^39^ The surface coatings are also expected to be protective layers for PET polymer from high temperature degradation without declining the flexibility of PET. Up to now, the method for completely avoiding the destruction of polymer substrates during the fabrication process of nanowire transparent electrodes by phonic sintering method has not been reported yet. In this study, we fabricated CuNW transparent electrodes on PET substrates with and without surface coatings. The morphology, transparency, and sheet resistance of the CuNW transparent electrodes were examined. The effect of coatings on the performance of the CuNW transparent electrodes was investigated.

## 2 Experimental

### 2.1 Synthesis of CuNWs

Analytical grade anhydrous copper dichloride (CuCl_2_), glucose, octadecylamine (ODA) were purchased from Wako Chemicals and used as received without further purification. CuNWs were synthesized by hydrothermal method as our previous papers.^28^ Firstly, 0.4 mmol CuCl_2_, 0.4 mmol glucose and 2.4 mmol ODA were mixed in 30 mL deionized water for 30 minutes under magnetic stirring until the solution turned into a blue emulsion. Then, the emulsion was kept at 120 °C in autoclave for 24 hours. Finally, the reddish product was collected and washed with water, hexane and isopropanol, respectively. The newly prepared CuNWs were dispersed in isopropanol to form Cu ink and stored in fridge before usage.

### 2.2 Fabrication of CuNW transparent electrodes

The Cu ink was treated with ultrasonic vibration for 15 minutes to achieve well-dispersed suspension. The Cu ink was sprayed onto PET substrates fixed on hot plate at 60 °C. In this study, two types of PET film were used as substrates for CuNW transparent electrodes. One type is bare PET, another type is commercial over head projection (OHP) film, which was PET polymer with surface coatings. The surface coatings were silicon oxides (SiO_2_) and aluminum oxide (Al_2_O_3_) nanoparticles, which will be characterized in detail later. We use N-PET and C-PET to name PET polymer without and with surface coatings as abbreviation, respectively. The amount of CuNWs on substrates was varied by changing spray time to obtain films with various transparencies. To achieve high conductivity, these CuNW films were treated with photonic sintering (PulseForge 3300, Novacentrix, Austin, TX, USA) at 552 mJ cm^−2^. The photonic sintering was operated at high voltage of 750 V and ten pulses (1 Hz) of light with extremely short duration of 30 μs were used to thoroughly sinter the CuNW films.

### 2.3 Characterization

The morphology of CuNW transparent electrode was characterized by field emission scanning electron microscopy (SEM, Hitachi SU8020, Hitachi High Technologies America, Inc.). The composition of the surface coatings was also investigated by X-ray diffractometry (XRD, Rigaku Smart Lab, Rigaku Americas Holding Company Inc.). The transmittance of CuNW transparent electrodes was measured using a UV-visible-near infrared spectrophotometer (V670, JASCO Corp.) at 550 nm. The sheet resistance was examined by the four-probe method with a surface resistivity meter (LorestaGPT610, Mitsubishi Chemical Analytech Co. Ltd). The bending test was examined using a machine which could bend the sample from 0° to 180° on rods with various diameters. The resistance was recorded in time using a resistance meter (RM 3544-01, Hioki E.E. Corporation). The thermal property of the PET films was tested by simultaneous thermogravimetry-differential scanning calorimetry, (STA/TG-DSC, STA449 F3, NETZCH) with a heating rate of 10 °C min^−1^ in nitrogen atmosphere.

## 3 Results and discussion

**Fig. 1:**
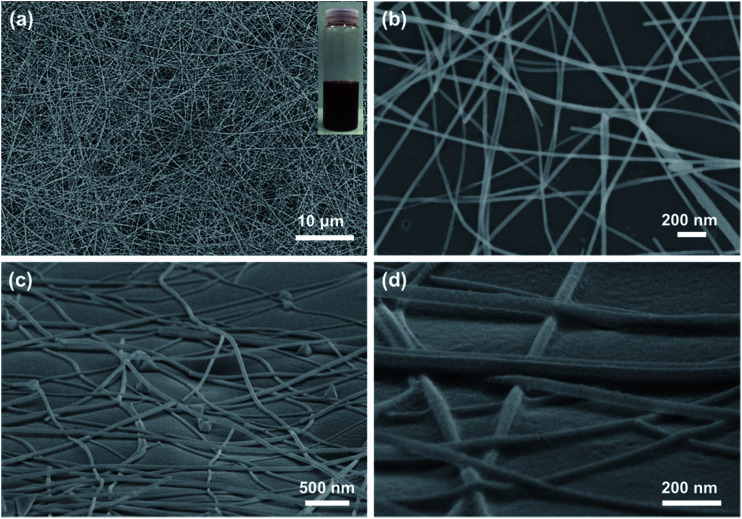
(a) SEM image of as-prepared CuNWs; top view (b) and tilted view (c and d) of CuNWs on bare N-PET substrates after photonic sintering. Inset in (a) shows the image of Cu ink.

Fig. 1(a) shows the morphology of as-synthesized CuNWs, which were with average diameter of 40 nm and length over 50 μm. The CuNWs were blended in isopropanol to make Cu ink (inset in Fig. 1(a)), which was in reddish color. Then the Cu ink was sprayed onto PET films to acquire homogeneously distributed CuNW network. The sheet resistance of the CuNW films without further treatment was around 10^5^ Ohm sq^−1^ or even larger. It was concluded the conductivity of metallic nanowire based films was mainly determined by the contact resistance between nanowires. Without other post-treatments, the contact between CuNWs was weak (Fig. S1†) and obstructed by the Cu oxides and organic residuals, leading to an extremely high sheet resistance for CuNW films. To improve the conductivity, the ink-printed PET films were treated by photonic sintering with light energy of 552 mJ cm^−2^ in air. In the present work, 10 pulses of intense light with duration of 30 μs was used to sinter the CuNW films. CuNW transparent electrodes were successfully achieved after the simple, rapid, and scalable photonic sintering. The morphology of CuNW transparent electrodes on N-PET film was given in Fig. 1(b)–(d). Fig. 1(b) shows the top view of CuNWs film with transmittance of 78%, showing random distribution of CuNWs. From the tilted view, the CuNWs formed a network structure by contacting with each other at the junctions on the slightly rolling N-PET film (Fig. 1(c)). It was found that the CuNWs were partly embedded into the surface of N-PET substrate (Fig. 1(d)). During the photonic sintering process, the upper parts of CuNWs were welded at junctions in air, meanwhile, the bottom parts were embedded in N-PET film, both of which were due to the thermal effect caused by the intense light. It was supposed that the high-energy light transformed into heat through the strongly adsorption of CuNWs. The generation of heat has two effects: (1) the CuNWs were heated to join with each other as in traditional thermal sintering process. As concluded in previous papers,^21,40^ the resistance of CuNW films was mainly determined by the contact resistance of junctions between CuNWs. The energy of highly intense light induced the diffusion and assembly of Cu atoms on the surface of nanowires, which contributed to a tight connection between CuNWs. Thus, the conductivity of CuNW films was obviously improved by enhancing the joining between CuNWs after the photonic sintering process. (2) The instantaneous high temperature caused by the light softened the surface of N-PET or even destroyed the polymer. From the close-up SEM image (Fig. 1(d)), the surface of N-PET was melted and partly adhered to the CuNWs. The enhanced adhesion between CuNWs and N-PET films was benefit to the mechanical robustness of the CuNW transparent electrodes. From above, the tightly connected CuNW network was generated and stuck on the flexible N-PET substrate after a rapid photonic sintering process.

**Fig. 2:**
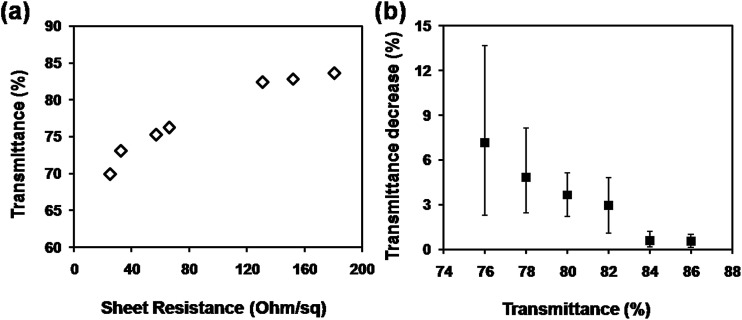
(a) Plot of transmittance *versus* sheet resistance for CuNW transparent electrodes on N-PET substrates; (b) transmittance decrease depending on the original transparency of CuNW electrodes.

Fig. 2 shows the plot of the transmittance at 550 nm *versus* sheet resistance for the CuNW transparent electrodes on N-PET substrates. In general, the sheet resistance was increased quickly when the transmittance was improved with less CuNWs on the N-PET substrates. CuNW transparent electrodes with sheet resistance of 131, 57, 25 Ohm sq^−1^ and transmittance of 82, 75, 70% were achieved after the photonic sintering process. During the photonic sintering process, the light energy transformed into heat energy, which induced the diffusion of Cu atoms and further enhanced the joining between CuNWs as shown in Fig. 1(d). Furthermore, the surface plasmon resonance (SPR) of CuNWs also focused the energy at the junctions promoting the connection between CuNWs. On the other hand, the ODA and Cu oxides were supposed to be removed by photochemical reactions as reported in our previous paper.^28^ Thus, the sheet resistance of the CuNW films was decreased from 10^5^ to less than 200 Ohm sq^−1^. Although high conductivity of CuNW films was greatly improved, the transmittance was decreased. Fig. 2(b) shows the transmittance decrease of CuNW electrodes after photonic sintering with light energy of 552 mJ cm^−2^ depending on the original transparency. It can be seen the transmittance decrease was greater when the original transparency was lower. The transmittance of 78%, 82% and 86% of CuNW films were decreased by 4.8%, 3.0% and 0.6% in average after photonic sintering, respectively. As mentioned above, the intense light transformed to heat that was supposed to damage the flexible N-PET polymer, causing lower transparency for CuNWs electrodes. When the CuNW films were placed under the xenon lamp, the light was absorbed by both CuNWs and N-PET substrates. The adsorption peak of N-PET was below 320 nm (Fig. S2†), which was sensitive to ultraviolet. Here, a quartz glass window was set between the lamp and the platform of the photonic sintering equipment, completely preventing the incidence of the ultraviolet light with wavelength below 200 nm. Even if the bare N-PET substrates were irradiated under same conditions, the transparency remained unchanged before and after the light sintering. However, the red-like CuNWs showed strong adsorption character at 580 nm in wavelength (Fig. S3†), which suggested that the radiant energy of the lamp could be adsorbed by the CuNWs. After light sintering, the substrate was warm, that meant the radiant energy of the lamp was converted into thermal energy to sinter the CuNWs. To investigate the heat production on the CuNW transparent electrodes, the temperature evolution was simulated by the software installed on the photonic sintering equipment using parameters as listed in Table S1 and S2.† The peak temperature on the surface of CuNWs network (with transmittance of 80%) was simulated to reach 365 °C within an extreme short duration of 30 μs (Fig. S4†). In contrast, the temperature at the bottom of the CuNW transparent electrodes was only increased to 53 °C thanking to the natural air-cooling system under the extremely short sintering. The metallic copper is with high thermal conductivity (401 W m^−1^ K^−1^), which could quickly transport the thermal energy to the interface between CuNWs and N-PET substrates. The heat was partly released to the bottom N-PET and warmed the PET to 53 °C. Most part of the heat was maintained at the interface due to the low thermal conductivity of PET (0.24 W m^−1^ K^−1^). Thus, the huge amount of heat at the interface softened or even melted the surface of N-PET to form a rugged surface structure, which decreased the transmittance of N-PET polymer (Fig. 2(b)). Moreover, the transmittance decline was related to the loading amount of CuNWs. The absorption intensity of CuNW network was proportional to the amount of CuNWs on PET. The more CuNWs were loaded on the PET, the more light was absorbed. The high loading CuNWs with lower original transmittance absorbed much more radiant lamp energy, leading to higher temperatures, which more seriously deformed the N-PET polymer. Thus, the transmittance of CuNW films with lower original transmittance declined more than that with higher original transmittance. From the above, although the photonic sintering method masterly welded the CuNWs network and helped in achieving highly conductive CuNW electrodes in a rather short processing duration, the excessive heat of radiant lamp deformed the N-PET substrate, resulting in considerable decrease in transmittance.

**Fig. 3:**
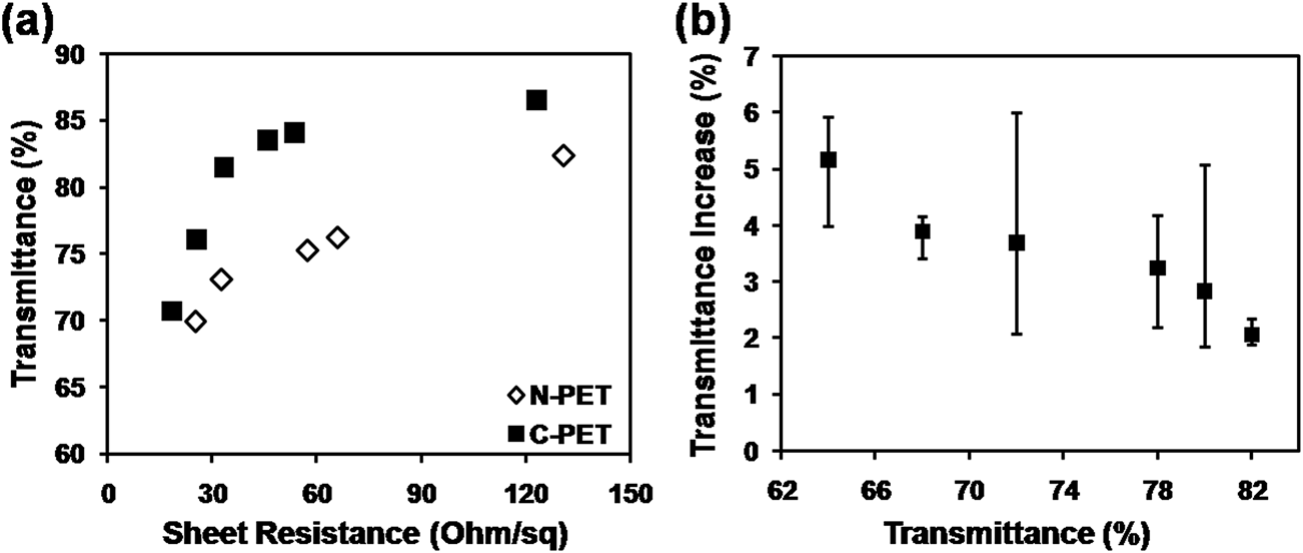
(a) Comparison of transmittance *versus* sheet resistance for CuNW transparent electrodes on N-PET and C-PET substrates; (b) transmittance increase depending on the original transparency of CuNW electrodes.

In order to fabrication of CuNW electrodes with both high conductivity and high transmittance by photonic sintering method, a commercial C-PET film was used to realize the target by avoiding the surface damage of PET with a surface thermal-barrier coating layer. Fig. 3 shows the properties of CuNW transparent electrodes after photonic sintering on C-PET compared with that on N-PET substrates. The sheet resistance was dramatically improved for the CuNW transparent electrodes on C-PET. For example, the CuNW electrode on C-PET substrates achieved a sheet resistance of 33 Ohm sq^−1^ with transmittance of 82%, which was much lower than that of CuNW electrodes on N-PET (131 Ohm sq^−1^). Moreover, the CuNW transparent electrodes on C-PET films exhibited excellent performance with sheet resistance of 25.5 Ohm sq^−1^ at transmittance of 76%, which was 25.1 Ohm sq^−1^ and 69% for N-PET substrates. It was indicated that the CuNW transparent electrodes on C-PET was superior to those films fabricated on N-PET substrate. This result indicates that the thermal-barrier coating layer on PET largely improved the conductivity and transparency of CuNWs film. On the other hand, it was found that the transmittance of CuNW transparent electrodes increased 1–6% rather than declined after photonic sintering (Fig. 3(b)). For example, the transmittance of CuNW films increased to 84% from 82% in average after 10 pulses of photonic sintering. It was indicated that the large amount of heat generated by the lamp gave rise to thermal stress in the CuNW network, which would break the network or separate CuNWs from the flat substrates. Moreover, the repeated heating supplied excessive energy in a short duration, causing huge thermal shock and blowing the CuNWs off the substrate.^28,41^ The detachment of CuNWs from substrates contributed to a higher transparency for CuNW transparent electrodes on C-PET as examined in Fig. 3(b). Thanks to the outstanding photonic sintering process, although small amount of CuNWs was detached from the C-PET, the CuNW electrodes still maintained high conductivity. Certainly, this phenomenon occurred during the fabrication of CuNW transparent electrodes on both N-PET and C-PET substrates. The N-PET polymer was severely degenerated under the highly intense pulsed light, which overshadowed the effect of detachment of CuNWs from N-PET substrates. Compared with N-PET, the coating layer on C-PET prevented the C-PET polymer from destroying by high-energy light. Thus, highly transparent CuNW electrodes were achieved by the versatile photonic sintering technique on commercial C-PET substrates by simply processing coating layer on bare PET polymer.

**Fig. 4:**
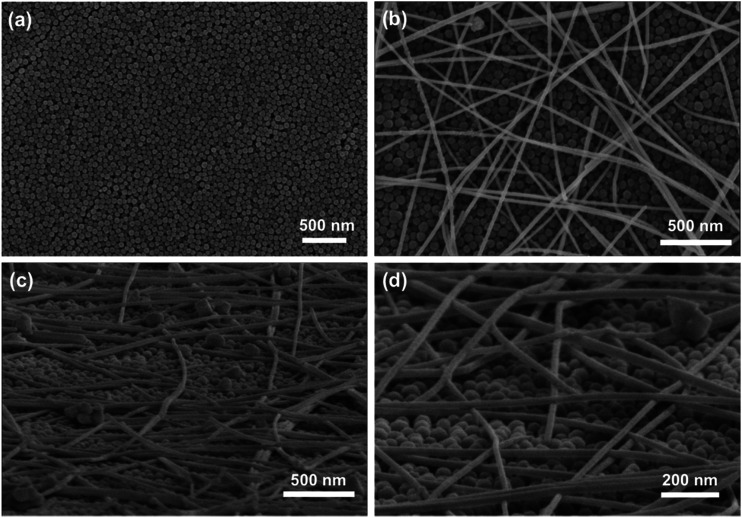
(a) SEM images of C-PET film; (b) top view and (c and d) tilted view of CuNWs on C-PET substrates.

To understand why the C-PET polymer avoided the destruction by photonic sintering, the morphologies of the CuNW transparent electrodes on C-PET substrates were examined. It was found that the surface of C-PET film was coated by uniform nanoparticles with size around 60 nm as shown in Fig. 4(a). The nanoparticles were proved to be SiO_2_ and Al_2_O_3_ combining the results of XRD and quantity analysis result using EDS (Fig. S5†). The CuNWs were stochastically scattered on the C-PET substrates as on N-PET film (Fig. 4(b)). From the tilted view of CuNW transparent electrodes on C-PET substrates in Fig. 4(c) and (d), the CuNWs were joined with each other at the cross points to form a huge network, lying on the flat C-PET polymer. To be mentioned, although the sizes of SiO_2_ and Al_2_O_3_ were extremely small, the nanoparticles were still well-defined without any melting or coalescing after photonic sintering, thanks to the high melting temperature of ceramic oxides. The melting point of SiO_2_ and Al_2_O_3_ was 1650 °C and 2054 °C, respectively, which was much higher than metal, let alone polymer. Even if the radiant lamp heated the surface of C-PET polymer, the coating on C-PET films was strong enough to withstand the high temperature sintering. The bare N-PET polymer was melted around 257 °C and decomposed at 428 °C (Fig. S6†). Nevertheless, the coating layer on PET was still stable up to 500 °C that the weight loss was 78% (for N-PET) for C-PET polymer indicating the presence of SiO_2_ and Al_2_O_3_ at 500 °C. The highly stable coating layer on C-PET polymer protected the substrate from direct exposure to the intense light. Furthermore, the heat rapidly transferred to the interface of CuNWs and C-PET polymer owing to the high thermal conductivity of the Cu metal, in contrast, only a small fraction of heat was released to the bottom part of C-PET substrate due to the low thermal conductivity of SiO_2_ and Al_2_O_3_. Thus, much more heat was remained at the interface of CuNWs and coating layer on C-PET substrates to improve the sintering of CuNWs. Hence, the C-PET polymer not only increased the conductivity of CuNWs but also avoided the destruction of PET even under high-energy light. The result implied that modification of the normal PET by surface coatings was crucial to obtain highly conductive CuNW transparent electrodes on flexible substrates. The only drawback is the additional coating of SiO_2_ and Al_2_O_3_ nanoparticles on the surface would slightly decrease the initial transmittance of PET polymer (Fig. S7†). Overall, it still opens a new way to optimize the rapid and simple photonic sintering technique for various flexible substrates.

**Fig. 5:**
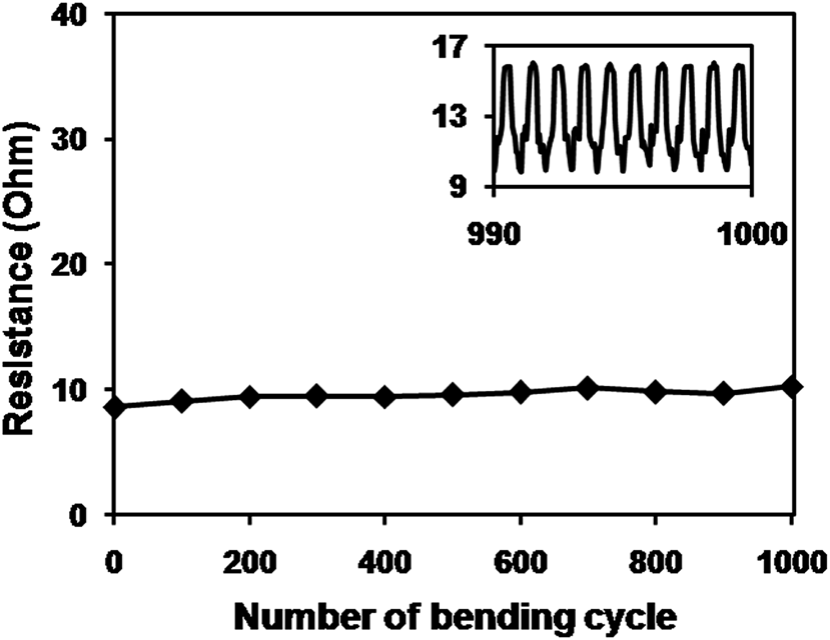
Resistance change of CuNW/PET transparent electrode during 1000 cycles of bending process. Inset graph shows the real-time resistance during the last ten cycles of bending and releasing process.

To check the flexibility of the CuNW transparent electrodes, the sintered CuNW film on C-PET substrate was bent for 1000 cycles on a rod with diameter of 5 mm (Fig. 5). A CuNW electrode with transparency of 76% and original resistance of 8.6 Ohm was clamped on the bending machine and connected with a resistance meter. The resistance of CuNW electrodes only increased to 10.3 Ohm even after 1000 cycles of bending and releasing, showing superior stability against deformation. Inset figure gives the real-time resistance curve during the last 10 cycles of bending test and no clear difference of resistance was observed after each bending. It was concluded that the intrinsic flexibility of CuNWs and the special network structure contributed to the good performance of CuNW transparent electrodes during the bending test. Respect for the excellent mechanical robustness, the CuNW transparent electrodes on C-PET substrates were expected to apply on other electronics, such as LED and solar cells and so on.

## 4 Conclusions

In summary, CuNW transparent electrodes were successfully achieved a simple and rapid photonic sintering method on surface-coated flexible PET substrates in ambient conditions without any protective atmosphere. The CuNWs were joined at the junctions thanks to the strong light absorption effect of CuNWs, which transferred the light energy to thermal energy to fuse nanowire together. The sheet resistance of CuNW transparent electrodes was 131 and 33.3 Ohm sq^−1^ at transmittance of 82% on N-PET and C-PET substrates, respectively. It was found that the excessive heat of radiant lamp deformed the N-PET substrate, resulting in considerable decrease in transmittance. Comparatively, the coating layer on the surface protected the C-PET from destroying by the instantaneous high temperature, which was benefit to keep a higher transparency for CuNW electrodes. Modification of flexible polymer film by various coatings would be an effective solution for fabrication of highly transparent CuNW electrodes by the versatile photonic sintering method and promoted the application of CuNW transparent electrodes on printed electronics.

## Conflicts of interest

There are no conflicts to declare.

## Supplementary Material

RA-008-C7RA12738C-s001

## References

[cit1] Wunscher S., Abbel R., Perelaer J., Schubert U. S. (2014). J. Mater. Chem. C.

[cit2] Im H. G., Jung S. H., Jin J., Lee D., Lee J., Lee D., Lee J. Y., Kim I. D., Bae B. S. (2014). ACS Nano.

[cit3] Ji S., Hyun B. G., Kim K., Lee S. Y., Kim S. H., Kim J. Y., Song M. H., Park J. U. (2016). NPG Asia Mater..

[cit4] Ji S., Jang J., Cho E., Kim S. H., Kang E. S., Kim J., Kim H. K., Kong H., Kim S. K., Kim J. Y., Park J. U. (2017). Adv. Mater..

[cit5] Kim M., Park J., Ji S., Shin S. H., Kim S. Y., Kim Y. C., Kim J. Y., Park J. U. (2016). Nanoscale.

[cit6] Wu J., Agrawal M., Becerril H. C. A., Bao Z., Liu Z., Chen Y., Peumans P. (2010). ACS Nano.

[cit7] Han S. T., Zhou Y., Chen B., Wang C., Zhou L., Yan Y., Zhuang J., Sun Q., Zhang H., Roy V. A. (2016). Small.

[cit8] Chu H. C., Chang Y. C., Lin Y., Chang S. H., Chang W. C., Li G. A., Tuan H. Y. (2016). ACS Appl. Mater. Interfaces.

[cit9] Su M., Li F., Chen S., Huang Z., Qin M., Li W., Zhang X., Song Y. (2016). Adv. Mater..

[cit10] Park J. H., Hwang G. T., Kim S., Seo J., Park H. J., Yu K., Kim T. S., Lee K. J. (2017). Adv. Mater..

[cit11] Bae S., Kim H., Lee Y., Xu X., Park J. S., Zheng Y., Balakrishnan J., Lei T., Kim H. R., Song Y. I., Kim Y. J., Kim K. S., Ozyilmaz B., Ahn J. H., Hong B. H., Iijima S. (2010). Nat. Nanotechnol..

[cit12] Lee J., Lee P., Lee H., Lee D., Lee S. S., Ko S. H. (2012). Nanoscale.

[cit13] Zhong Z., Woo K., Kim I., Hwang H., Kwon S., Choi Y. M., Lee Y., Lee T. M., Kim K., Moon J. (2016). Nanoscale.

[cit14] Hong S., Yeo J., Kim G., Kim D., Lee H., Kwon J., Lee H., Lee P., Ko S. H. (2013). ACS Nano.

[cit15] Hellstrom S. L., Lee H. W., Bao Z. (2009). ACS Nano.

[cit16] Lipomi D. J., Vosgueritchian M., Tee B. C., Hellstrom S. L., Lee J. A., Fox C. H., Bao Z. (2011). Nat. Nanotechnol..

[cit17] Wu Z., Chen Z., Du X., Logan J. M., Sippel J., Nikolou M., Kamaras K., Reynolds J. R., Tanner D. B., Hebard A. F., Rinzler A. G. (2004). Science.

[cit18] Hwang J. O., Park J. S., Choi D. S., Kim J. Y., Lee S. H., Lee K. E., Kim Y.-H., Song M. H., Yoo S., Kim S. O. (2012). ACS Nano.

[cit19] Liu Z., You P., Xie C., Tang G., Yan F. (2016). Nano Energy.

[cit20] Jo G., Choe M., Cho C. Y., Kim J. H., Park W., Lee S., Hong W. K., Kim T. W., Park S. J., Hong B. H., Kahng Y. H., Lee T. (2010). Nanotechnology.

[cit21] Lee J., Lee I., Kim T. S., Lee J. Y. (2013). Small.

[cit22] Tokuno T., Nogi M., Karakawa M., Jiu J., Nge T. T., Aso Y., Suganuma K. (2011). Nano Res..

[cit23] Hu L., Kim H. S., Lee J.-Y., Peumans P., Cui Y. (2010). ACS Nano.

[cit24] Guo H., Lin N., Chen Y., Wang Z., Xie Q., Zheng T., Gao N., Li S., Kang J., Cai D., Peng D. L. (2013). Sci. Rep..

[cit25] Rathmell A. R., Wiley B. J. (2011). Adv. Mater..

[cit26] Ye S., Rathmell A. R., Stewart I. E., Ha Y. C., Wilson A. R., Chen Z., Wiley B. J. (2014). Chem. Commun..

[cit27] Jiu J., Nogi M., Sugahara T., Tokuno T., Araki T., Komoda N., Suganuma K., Uchida H., Shinozaki K. (2012). J. Mater. Chem..

[cit28] Ding S., Jiu J., Tian Y., Sugahara T., Nagao S., Suganuma K. (2015). Phys. Chem. Chem. Phys..

[cit29] Jiu J., Sugahara T., Nogi M., Araki T., Suganuma K., Uchida H., Shinozaki K. (2013). Nanoscale.

[cit30] Joo S. J., Park S. H., Moon C. J., Kim H. S. (2015). ACS Appl. Mater. Interfaces.

[cit31] Hwang Y. T., Chung W. H., Jang Y. R., Kim H. S. (2016). ACS Appl. Mater. Interfaces.

[cit32] Li W., Zhang H., Gao Y., Jiu J., Li C.-F., Chen C., Hu D., Goya Y., Wang Y., Koga H., Nagao S., Suganuma K. (2017). J. Mater. Chem. C.

[cit33] Yılmaz Ş., Ipek M., Celebi G. F., Bindal C. (2005). Vacuum.

[cit34] Sarikaya O. (2005). Mater. Des..

[cit35] Kumar V., Balasubramanian K. (2016). Prog. Org. Coat..

[cit36] Loghman-Estarki M. R., Shoja Razavi R., Edris H., pourbafrany M., Jamali H., ghasemi R. (2014). Ceram. Int..

[cit37] Moridi A., Azadi M., Farrahi G. H. (2014). Surf. Coat. Technol..

[cit38] Singh V. P., Sil A., Jayaganthan R. (2011). Mater. Des..

[cit39] Wang M. J., Li C. F., Yen S. K. (2013). Corros. Sci..

[cit40] Won Y., Kim A., Lee D., Yang W., Woo K., Jeong S., Moon J. (2014). NPG Asia Mater..

[cit41] Li W., Hu D., Li L., Li C.-F., Jiu J., Chen C., Ishina T., Sugahara T., Suganuma K. (2017). ACS Appl. Mater. Interfaces.

