# Supplementary material for: Highly conductive and transparent copper nanowire electrodes on surface coated flexible and heat-sensitive substrates
Source: RSC Adv. 2018 Jan 9;8(4):2109–15. doi: 10.1039/c7ra12738c (PMC9077247; doi:10.1039/c7ra12738c)
Supplement: RA-008-C7RA12738C-s001 [file RA-008-C7RA12738C-s001.pdf]

Electronic Supplementary Material (ESI) for RSC Advances.
This journal is © The Royal Society of Chemistry 2017

Supplementary Information

Highly conductive and transparent copper nanowire electrodes on surface coated flexible and heat-sensitive substrates

Su Ding[a,b], Yanhong Tian[a,*], Jinting Jiu[c,*], Katsuaki Suganuma[c]

[a]State Key Laboratory of Advanced Welding and Joining, Harbin Institute of Technology, Harbin, 150001, China

[b]College of Materials and Environmental Engineering, Hangzhou Dianzi University, Hangzhou, 310036, China

[c]The Institute of Scientific and Industrial Research (ISIR), Osaka University, Osaka 5650871, Japan

Corresponding authors: tianyh@hit.edu.cn, jiu@eco.sanken.osaka-u.ac.jp

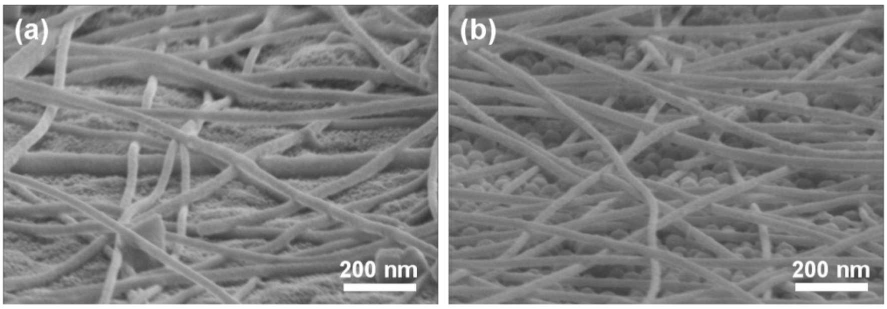

Fig. S1 Tilted SEM images of CuNW networks on N-PET and C-PET, respectively

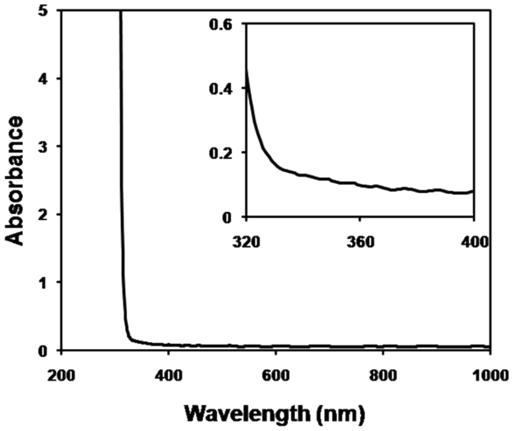

Fig. S2 Adsorption spectrum of N-PET polymer.

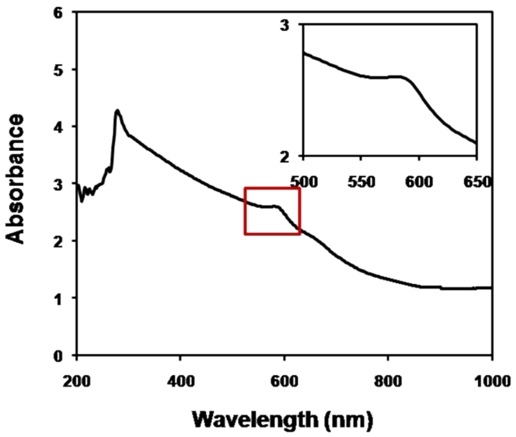

Fig. S3 Adsorption spectrum of CuNWs.

Tab. S1 Parameters of Cu and N-PET used in the simulation

**Material name**	**Thermal conductivity (W/mK)**	**Mass density (g/cm^3)**	**Specific heat (J/kg)**	**Melt temperature (Degree)**	**Absorptivity (%)**
**Cu**	401	8.96	382.5	1084.6	20
**N-PET**	0.24	1.4	730	370	-

Tab. S2 Parameters of the photonic sintering machine

**Voltage (V)**	**Pulse duration (μs)**	**Pulse number**	**Pulse frequency (Hz)**
750	30	10	1

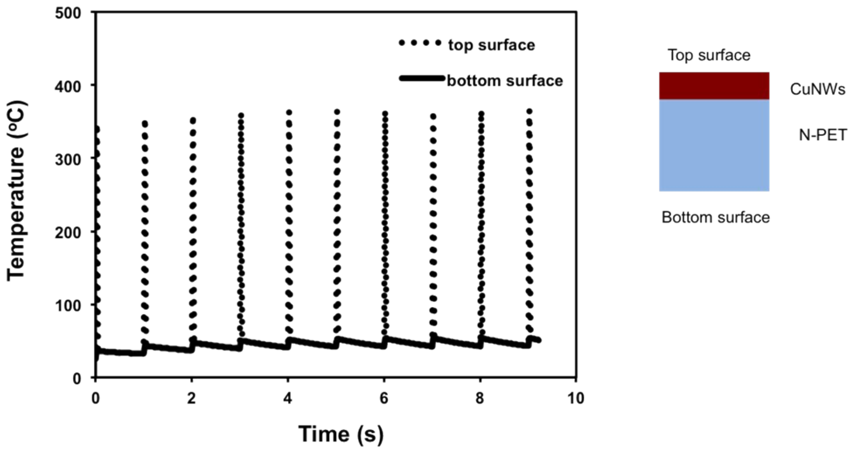

Fig. S4 Simulation result of the temperature evolution at the surface and the bottom of the CuNW transparent electrodes on N-PET substrate.

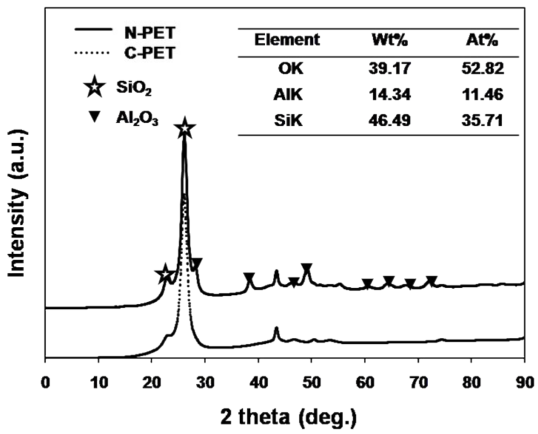

Fig. S5 XRD curves of N-PET and C-PET polymer. Inset is the quantity analysis result using energy dispersive spectrometer (EDS) result of C-PET.

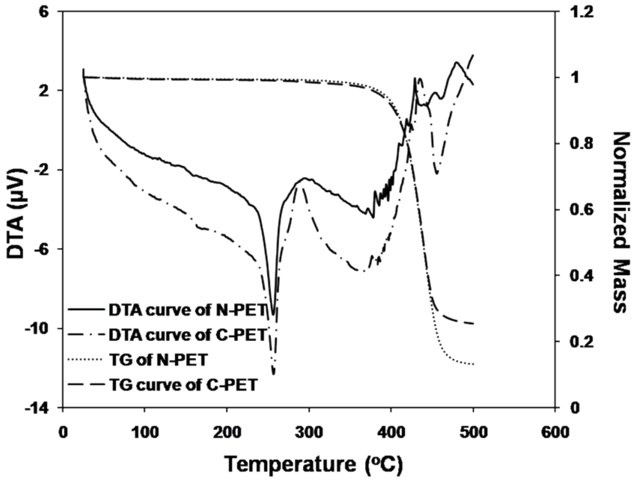

Fig. S6 DTA/TG curves of N-PET and C-PET polymer.

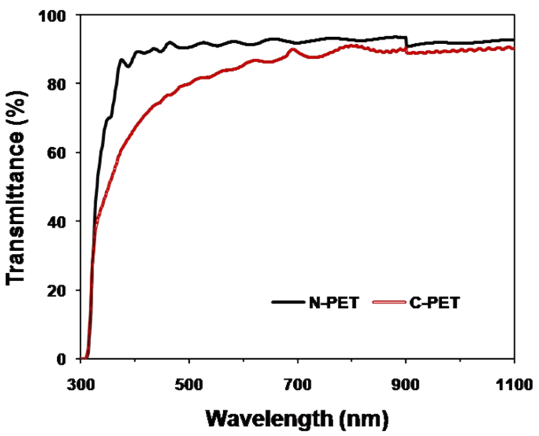

Fig. S7 Comparison of transmittance curves for bare N-PET and C-PET.
